# Software-Defined Doppler Radar Sensor for Human Breathing Detection

**DOI:** 10.3390/s19143085

**Published:** 2019-07-12

**Authors:** Sandra Costanzo

**Affiliations:** DIMES, Università della Calabria, 87036 Rende, Italy; costanzo@dimes.unical.it

**Keywords:** microwave sensors, software-defined radar, biomedical applications

## Abstract

Non-contact wireless sensing approaches have emerged in recent years, in order to enable novel enhanced developments in the framework of healthcare and biomedical scenarios. One of these technologically advanced solutions is given by software-defined radar platforms, a low-cost radar implementation, where all operations are implemented and easily changed via software. In the present paper, a software-defined radar implementation with Doppler elaboration features is presented, to be applied for the non-contact monitoring of human respiration signals. A quadrature receiver I/Q (In-phase/Quadrature) architecture is adopted in order to overcome the critical issues related to the occurrences of null detection points, while the phase-locked loop components included in the software defined radio transceiver are successfully exploited to guarantee the phase correlation between I/Q signal components. The proposed approach leads to a compact, low-cost, and flexible radar solution, whose application abilities may be simply changed via software, with no need for hardware modifications. Experimental results on a human target are discussed so as to demonstrate the feasibility of the proposed approach for vital signs detection.

## 1. Introduction

Rapid advances in wireless technologies now make the implementation of compact, lightweight, and highly integrated systems feasible, in order to improve life quality through the early diagnosis and continuous non-invasive monitoring of physiologic parameters [1].

Radar-based techniques have been historically employed (since the late 1970s) to detect human vital signs, such as respiration and heartbeat, by analyzing the interaction between radiofrequency signals and physiological movements, without requiring any contact with the human body [2].

In particular, Doppler radar sensing devices [3,4,5] transmit a continuous wave (CW) signal, which is reflected off the human target and is successfully demodulated at the receiver stage to provide a signal proportional to the target oscillation. If analyzing the received signal, a time delay with respect to the transmitted wave can be identified. It is determined by both the nominal distance (*d_o_*) of the target, as well as by a phase function modulated by the periodic motion (*x(t)*) of the target. The information about the oscillation movement (*x(t)*) is retrieved (demodulated) by multiplying the received signal with the local oscillator (LO) signal, and then low-pass filtering the result to obtain a useful baseband signal. When assuming a human subject as target, the heartbeat and breathing movements can be extracted from the baseband signal, which is a function of the periodic oscillation (*x(t)*).

However, some critical points, concerning the presence of a residual phase noise and the occurrences of null detection points (depending on the nominal human target distance (*d_o_*)), should be properly managed in order to guarantee the accurate detection of vital signs [6]. In the present paper, the above critical issues are both eliminated by adopting a software-defined radar (SDRadar) architecture, which provides a simple, low-cost, and flexible alternative to standard bulky radar systems.

First introduced by the author of [7], the SDRadar concept leads to a new radar paradigm, where all functions are fully implemented via software, thus providing a solution to design customized radar systems, able to operatively modify their abilities with no change in the hardware configuration. The capabilities of the SDRadar approach have been illustrated in the literature [7,8,9,10], and its applicability to vibrations detection was been first demonstrated by the authors of [11,12], and recently has also been explored by the authors of [13,14].

In the present paper, a Doppler SDRadar architecture is illustrated, to be applied for the non-contact detection of human respiration signals. A specific I/Q configuration, based on the adoption of a quadrature receiver [15], is considered to overcome problems related to the occurrence of null detection points, while the inclusion of phase-locked (PLL) components in the software defined radio (SDR) transceiver is exploited so as to guarantee the phase correlation of the I/Q signals, thus avoiding the presence of undesirable residual phase noise terms. The measurement tests and experimental validation results on human subjects are reported and discussed. 

## 2. Software-Defined Doppler Radar Principle

**Figure 1:**
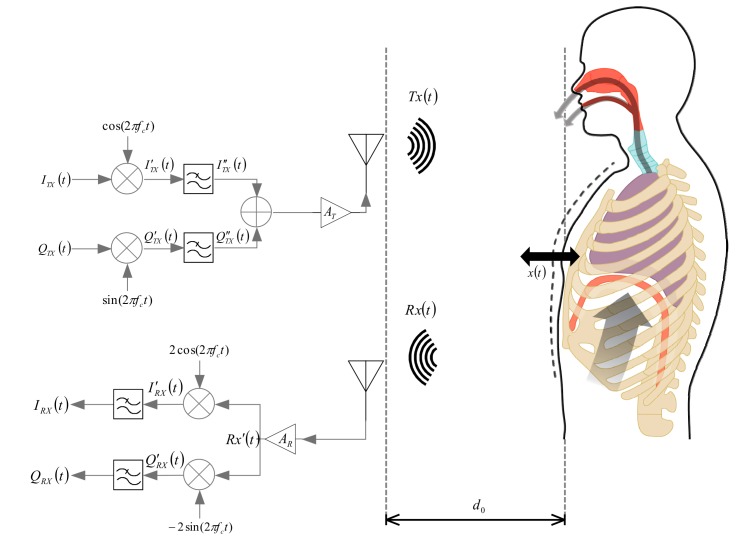
A continuous wave (CW) Doppler radar platform.

The proposed SDRadar platform, based on the adoption of a quadrature receiver architecture, is illustrated in Figure 1.

A CW narrow-band signal is typically transmitted; it is reflected off the human target and subsequently demodulated in the homodyne or heterodyne receiver. With reference to the above scenario, two parameters can be specifically considered in order to characterize the alternate motion associated with human respiration, namely the time-varying signal (*x(t)*), modeling the human oscillations as a result of the vital sign, and the target range (*d_o_*), giving the distance around which the oscillations occur.

A completely new SDRadar configuration is assumed, as compared to the developments illustrated in the first work [11], where a single-channel receiver was considered, thus limiting the accuracy detection to the optimum detection points (target distance *d_o_* = λ(1/8 + *k* × 1/4); *k* is the integer), while inhibiting the application to the null detection points (*d_o_* = λ(1/4 + *k* × 1/4), *k* integer). In the present work, a quadrature receiver configuration, with separate signal processing operations (i.e., modulation, mixing, and filtering), is considered, thus leading to reconstruct vibrations for arbitrary target distances (*d_o_* < λ, λ) being the wavelength associated to the CW transmitted frequency (*f_o_*). Possible interferences because of other wireless devices should preserve the condition that the resulting oscillation amplitude will be less than the above wavelength (λ), in order to apply the small angle approximation for reconstructing the target oscillation motion.

To generate the monochrome CW signal, the following in-phase (I) and in-quadrature (Q) components need to be fixed:

(1) $$I_{TX}\left(t\right)=\cos\left[2\pi f_{\mathrm{IF}}t+\theta_{\mathrm{IF}}\left(t\right)\right]$$

(2) $$Q_{TX}\left(t\right)=\sin\left[2\pi f_{\mathrm{IF}}t+\theta_{\mathrm{IF}}\left(t\right)\right]$$

where parameters *f*_IF_ and *θ*_IF_ give the intermediate frequency and the phase noise, respectively.

In the SDRadar platform, the I/Q components are applied to different modulator branches and are subject to mixing, filtering, and sum and amplification operations, shifting their signal spectrum from the intermediate frequency (f_IF_) to the carrier frequency (fc-f_IF_) for the up-conversion operation.

The backscattered I/Q components, received from the radar, keep the same form as the transmitted ones, but they are attenuated of a factor, taking into account the radar cross section of the target and the propagation losses, and they are also delayed by the so-called “round trip” time *τ*(t), given as [16] the following:

(3) $$\tau \left(t\right)=\frac{2d_{0}}{c}+\frac{2x\left(t\right)}{c}$$

The extraction of the received I/Q components is performed by dividing the received signal and assigning it in parallel to the two demodulator branches. Through mixing and filtering operations, the spectrum of the two signal components is moved once again from the carrying frequency (fc-f_IF_) to the intermediate frequency (f_IF_), thus performing the down-conversion processing.

**Figure 2:**
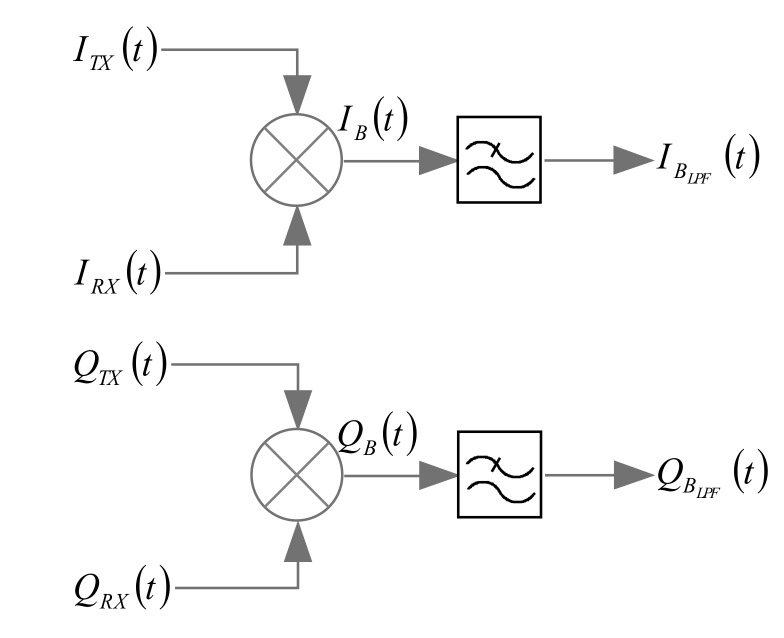
I/Q component extraction for phase elaboration.

The information about the monitored target oscillation (*x(t)*) is retrieved from the phase of the received signal, which in turns is extracted according to the scheme illustrated in Figure 2.

In particular, the transmitted and the received signals (suffixes Tx and Rx, respectively, in Figure 2) are mixed and low-pass filtered, thus obtaining the following I/Q components [16]:

(4) $$I_{B_{LPF}}\left(t\right)=\frac{A}{2}\cos\left[-\frac{4\pi \left(f_{c}-f_{IF}\right)d_{0}}{c}-\frac{4\pi \left(f_{c}-f_{IF}\right)x\left(t\right)}{c}+\Delta \theta_{TX}\left(t\right)\right]$$

(5) $$Q_{B_{LPF}}\left(t\right)=\frac{A}{2}\sin\left[-\frac{4\pi \left(f_{c}-f_{IF}\right)d_{0}}{c}-\frac{4\pi \left(f_{c}-f_{IF}\right)x\left(t\right)}{c}+\Delta \theta_{TX}\left(t\right)\right]$$

where the term A/2 gives the overall attenuation factor, and the delay expressed by Equation (3) is also included.

Because of the presence of PLL components in the SDRadar architecture, the residual phase noise (Δ*θ*_TX_) can be neglected, thus leading to writing the following (from combination of Equations (4) and (5)):

(6) $$\arctan\left[\frac{Q_{B_{LPF}}\left(t\right)}{I_{B_{LPF}}\left(t\right)}\right]=\left[-\frac{4\pi \left(f_{c}-f_{IF}\right)d_{0}}{c}-\frac{4\pi \left(f_{c}-f_{IF}\right)x\left(t\right)}{c}\right]$$

Finally, from the inversion of Equation (6), it is possible to easily derive the target oscillation, as follows:

(7) $$\bar{x}\left(t\right)=d_{0}+x\left(t\right)=-\arctan\left[\frac{Q_{B_{LPF}}\left(t\right)}{I_{B_{LPF}}\left(t\right)}\right]\frac{c}{4\pi \left(f_{c}-f_{IF}\right)}$$

**Figure 3:**
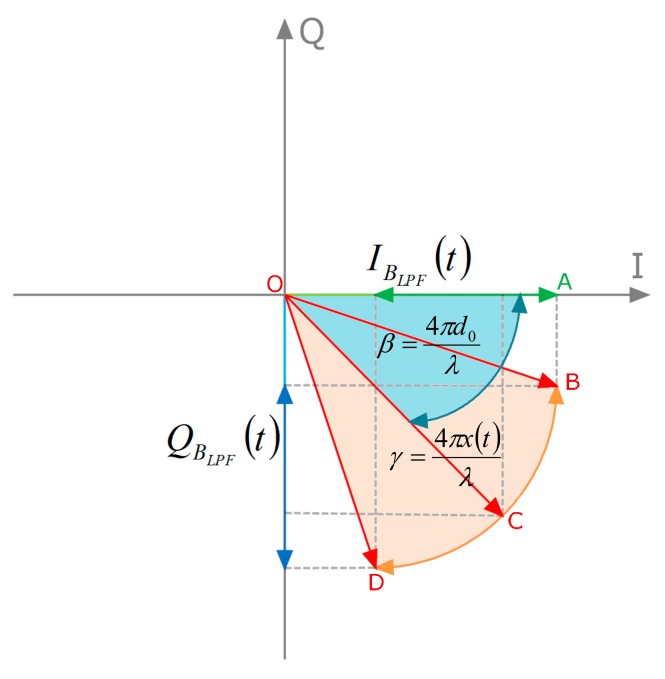
Complex plane representation of Equation (7).

A geometric illustration of Equation (7) is shown in Figure 3, where the complex plane representation identifies a vector (*OC*) rotating within the area (*OB-OD*), to give the phase term (*γ*) linearly dependent on the target motion (*x(t)*).

## 3. Software-Defined Doppler Radar Architecture

**Figure 4:**
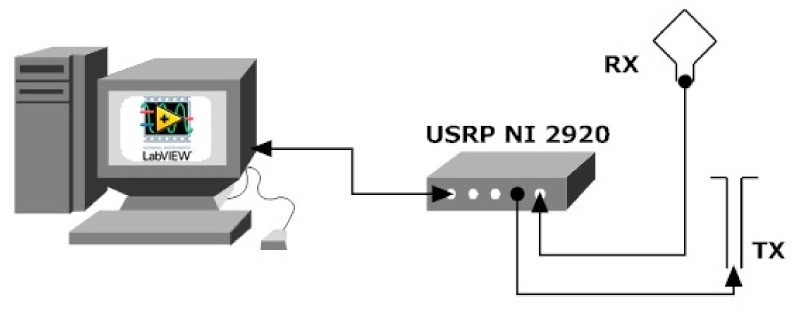
Software-defined radar (SDRadar) schematic architecture.

As outlined in the Introduction, an innovative Doppler radar configuration, based on the SDRadar paradigm, is adopted as an alternative to a standard hardware platform, and is applied for the accurate detection of small oscillations, such as those encountered in the framework of human vital signs identification. The SDRadar architecture, illustrated in Figure 4, is extremely simple, and it basically consists of four main blocks, namely:

- a general-purpose PC, simply equipped to support the software for the connection to the SDR transceiver and the signal processing operations;
- the SDR transceiver (NI USRP-2920, in the present implementation);
- the transmitting (T_x_) and receiving (R_x_) antenna.

**Figure 5:**
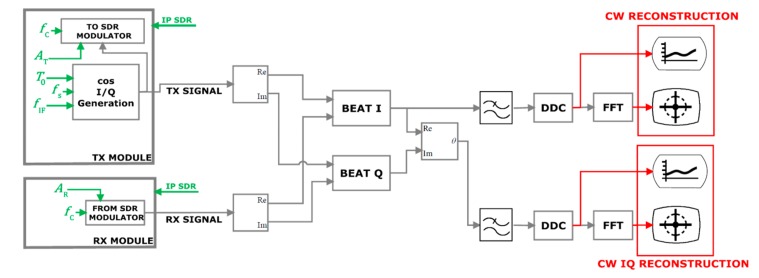
Software architecture of SDRadar with Doppler elaborations.

The main core of the SDRadar platform is represented by the SDR transceiver, enabling the software implementation of all radar operations, namely, the signal generation, filtering, mixing, modulation/demodulation, and data processing. In our implementation, the adopted NI USRP-2920 (Impinj, Inc., Seattle, WA, USA), is able to work in a frequency range going from 50 MHz up to 2.2. GHz, with a 20 MHz bandwidth and a dynamic range equal to 80 dB; it includes two physical channels (Figure 5) for the transmission and the reception paths, and it is equipped with an I/Q modulator and demodulator, enabling the adoption of a quadrature receiver, which overcomes problems related to null detection points. As a further advantageous feature, the SDR transceiver is equipped with two oscillators, including PLL components, thus leading to neglecting the residual phase noise terms in the baseband modulated phase.

To properly realize the accurate detection of human oscillations, described in Section 2, the software architecture is properly improved beyond the standard NI commercial product, as illustrated in Figure 5.

- a real time set of parameters for signal generation (green elements in Figure 5), fixing in turn the radar resolutions;
- the illustrations of the elaboration results (red elements in Figure 5).

In particular, a LabVIEW user interface is implemented to allow the operator to do the following.

A specific acquisition form leads the user to set the relevant parameters, such as the carrier frequency, the bandwidth, and the T_x_ and R_x_ gains, which in turn determine the radar range. The Doppler elaboration feature is specifically implemented, thus providing the speed resolution, frequency resolution, and maximum attainable speed.

From the conceptual point of view, the proposed architecture is inherently a coherent low-IF Doppler radar [17], typically adopted in the literature to overcome the issues and limitations inherent to the direct conversion receivers and phase correlation problems of the transmit and receive local oscillators. However, when compared to the existing hardware configurations, the main relevance of the proposed architecture is related to the software-defined approach, which leads to the implementation of a dual-receiver configuration via software to be very complicated if considered from the hardware point of view. With the above software approach, the operating frequency of the radar system, and the related bandwidth and resolutions, can be easily changed with no modification in the hardware structure, thus leading to a low-cost and extremely flexible radar solution. Furthermore, the presence of PLL components, included in the SDR transceivers, automatically avoids residual phase noise terms, usually limiting the performances of standard configurations.

## 4. Experimental Validations

**Figure 6:**
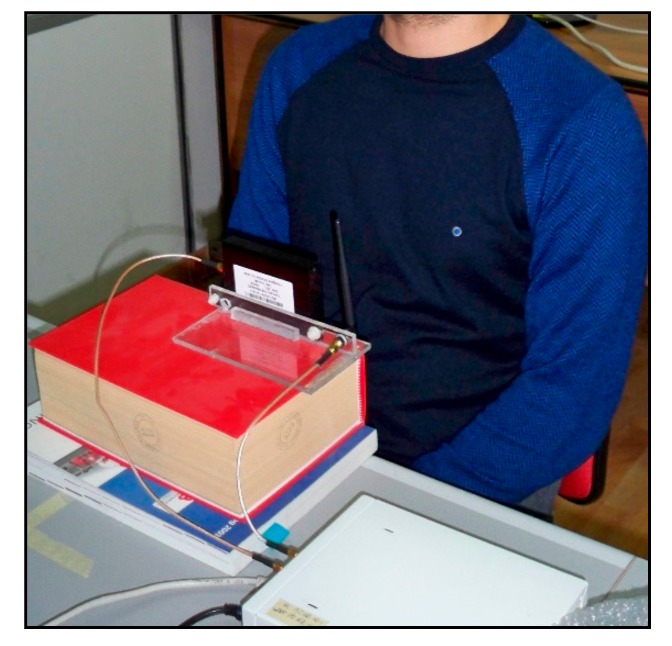
Experimental setup for the monitoring of the respiration activity.

Useful experimental validations on human subjects are performed so as to assess the effectiveness of the proposed SDRadar platform for the identification of breath signals. To this end, the experimental setup, illustrated in Figure 6, is considered, where the T_x_ and R_x_ antennas (a vertical dipole antenna and a omnidirectional antenna Impinj A0303 (Impinj, Inc., Seattle, WA, USA), respectively) are placed in front of a human chest. For the currently adopted antennas, a maximum distance of the radar equal to 80 cm can be considered to achieve accurate performances.

**Figure 7:**
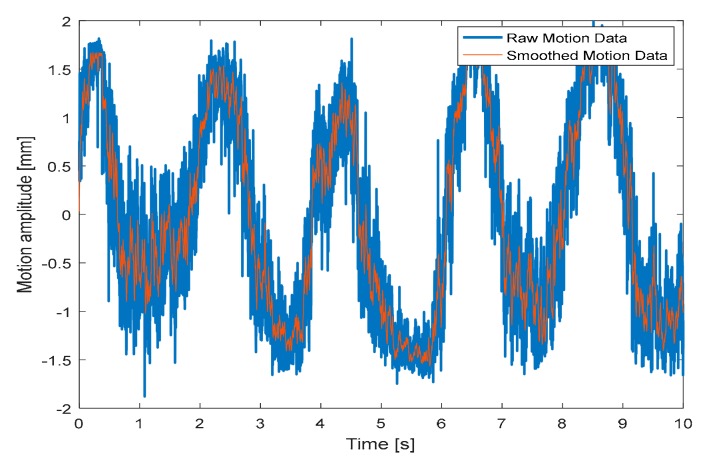
Detected movement (*x(t*)*)* under normal breath.

**Figure 8:**
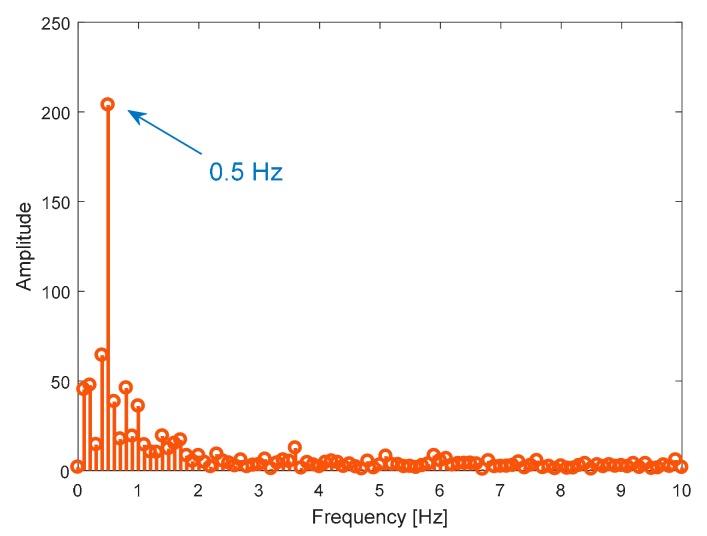
Spectral analysis of detected movement in Figure 7.

Experimental tests for the monitoring of human target breath are performed under three different conditions, namely normal, accelerated, and interrupted respiration. The detected movement under normal breath (Figure 7) reveals regular behavior, with an identified abdominal shift of about 3 mm. To refine the reconstruction, a post elaboration filtering, namely the moving average filter in a MATLAB environment, is applied, thus smoothing the original captured data. The spectral analysis reported in Figure 8 leads to identifying an oscillation frequency equal to 0.5 Hz, which is equivalent to a human breath rate of 30 breaths/min.

**Figure 9:**
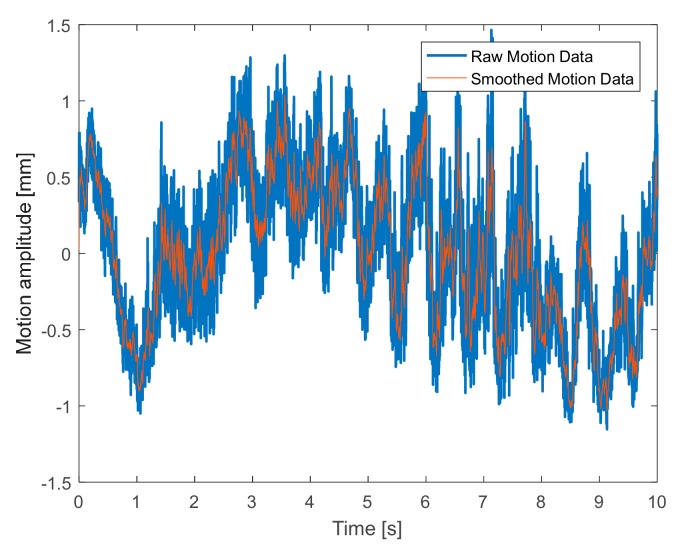
Detected movement (*x(t)*) under accelerated breath.

**Figure 10:**
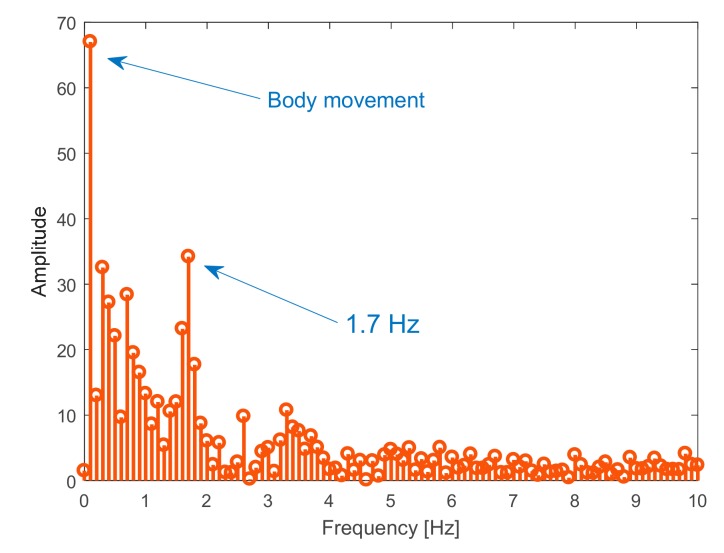
Spectral analysis of detected movement in Figure 9.

When imposing the accelerated respiration activity, an irregular movement, illustrated in Figure 9, was detected, whose corresponding spectrum is reported in Figure 10. In particular, this reveals a slight body movement partially influencing the measurements, and an oscillation frequency equal to 1.2 Hz, which corresponds to a human breath rate of 102 breaths/min.

**Figure 11:**
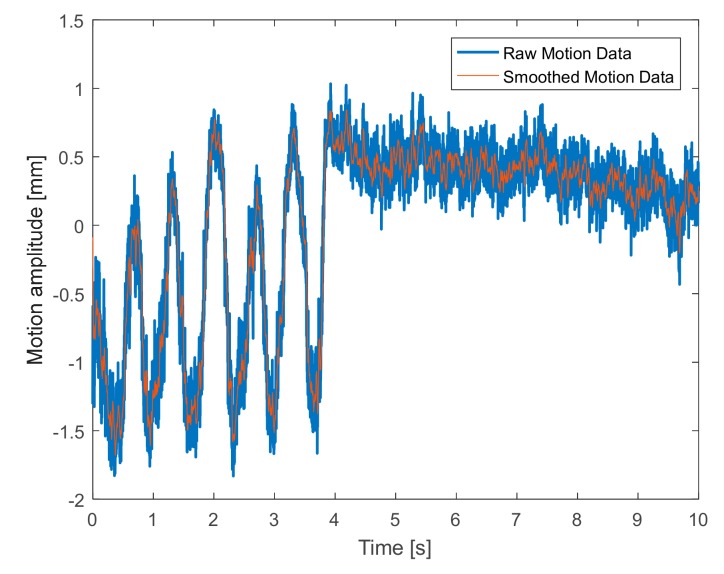
Detected movement (*x(t)*) under normal/interrupted breath.

Finally, when forcing an interrupted respiration activity, the time behavior illustrated in Figure 11 is retrieved, leading to easily distinguishing between the two states of normal respiration and interrupted respiration.

## 5. Conclusions

An SDRadar platform with Doppler elaboration features has been presented in this paper, to be applied for the non-contact monitoring of low-frequency oscillations typical of human breath. Both hardware and software architectures of the enhanced radar configuration have been described, whose main advantages can be summarized as follows:

- the ability to implement and real-time change via software all radar functions, without any hardware modification;
- availability of an I/Q extraction feature, leading to adopting a quadrature receiver, which avoids the occurrences of null detection points, typical encountered in standard CW radar configurations;
- availability of PLL components, leading to neglecting the residual phase noise terms in the modulated phase, giving the oscillation function.

A full implementation of the proposed SDRadar system has been described, by adopting an NI USRP-2920 transceiver and LabVIEW software platform to implement a specific user interface for the radar control and data processing. Experimental validations on a human subject have been discussed so as to prove the ability of the proposed enhanced radar platform to accurately detect human breath.
